# Advances in Laser Additive Manufacturing of Ti-Nb Alloys: From Nanostructured Powders to Bulk Objects

**DOI:** 10.3390/nano11051159

**Published:** 2021-04-29

**Authors:** Margarita A. Khimich, Konstantin A. Prosolov, Tatiana Mishurova, Sergei Evsevleev, Xavier Monforte, Andreas H. Teuschl, Paul Slezak, Egor A. Ibragimov, Alexander A. Saprykin, Zhanna G. Kovalevskaya, Andrey I. Dmitriev, Giovanni Bruno, Yurii P. Sharkeev

**Affiliations:** 1 Laboratory of Nanobioengineering, Laboratory of Nanostructured Biocomposites, Laboratory of Computer-Aided Design of Materials, Institute of Strength Physics and Materials Science of SB RAS, 2/4, Akademicheskii pr., 634055 Tomsk, Russia; khimich@ispms.ru (M.A.K.); konstprosolov@gmail.com (K.A.P.); sharkeev@ispms.ru (Y.P.S.); 2 Physics Technical Faculty, Tomsk Material Science Common Use Center, National Research Tomsk State University, 36, Lenina pr., 634050 Tomsk, Russia; 3 Department of Non-Destructive Testing, Division 8.5 Micro NDE, Bundesanstalt für Materialforschung und -Prüfung (BAM), Unter den Eichen 87, 12205 Berlin, Germany; tatiana.mishurova@bam.de (T.M.); sergei.evsevleev@bam.de (S.E.); giovanni.bruno@bam.de (G.B.); 4 Department of Life Science Engineering, University of Applied Sciences Technikum Wien, Höchstädtpl. 6, 1200 Vienna, Austria; monforte@technikum-wien.at (X.M.); andreas.teuschl@technikum-wien.at (A.H.T.); 5 Ludwig Boltzmann Institute for Experimental and Clinical Traumatology, AUVA Research Center, Donaueschingenstraße 13, 1200 Vienna, Austria; Paul.Slezak@trauma.lbg.ac.at; 6 Material Science Department, Research School of Physics of High Energy Processes, National Research Tomsk Polytechnic University, Yurga Technical University TPU Affiliate, 30, Lenina pr., 634050 Tomsk, Russia; egor83@list.ru (E.A.I.); sapraa@tpu.ru (A.A.S.); kovalevskaya@tpu.ru (Z.G.K.); 7 Institute of Physics and Astronomy, University of Potsdam, Karl-Liebknecht-Str. 24-25, 14476 Potsdam, Germany

**Keywords:** additive manufacturing, biomaterials, Ti-Nb alloy, nanostructured powder, laser methods, powder methods, laser powder bed fusion

## Abstract

The additive manufacturing of low elastic modulus alloys that have a certain level of porosity for biomedical needs is a growing area of research. Here, we show the results of manufacturing of porous and dense samples by a laser powder bed fusion (LPBF) of Ti-Nb alloy, using two distinctive fusion strategies. The nanostructured Ti-Nb alloy powders were produced by mechanical alloying and have a nanostructured state with nanosized grains up to 90 nm. The manufactured porous samples have pronounced open porosity and advanced roughness, contrary to dense samples with a relatively smooth surface profile. The structure of both types of samples after LPBF is formed by uniaxial grains having micro- and nanosized features. The inner structure of the porous samples is comprised of an open interconnected system of pores. The volume fraction of isolated porosity is 2 vol. % and the total porosity is 20 vol. %. Cell viability was assessed in vitro for 3 and 7 days using the MG63 cell line. With longer culture periods, cells showed an increased cell density over the entire surface of a porous Ti-Nb sample. Both types of samples are not cytotoxic and could be used for further in vivo studies.

## 1. Introduction

It is now possible to manufacture products made of a variety of different materials, such as ceramics, polymers, and metals. Titanium-based alloys are widely used in the field of medical implant production [1] due to their biocompatibility and good physical and mechanical properties [2,3,4,5,6]. The excellent biocompatibility of commercially pure titanium (c.p. Ti) is associated with the ability of this material to form a thin oxide layer on its surface that provides its bioinert property and corrosion resistance [7,8,9,10,11]. In the case of hip replacement implants (HRI), products that are made of Ti-based alloys consist of a scaffold designed to replace a segment of load-bearing bone. It is of vital importance for HRI to have sufficient strength and long fatigue life [12]. At present, the majority of industrially used titanium-based alloys contain elements such as Al, V, Fe, Ni, Co, and Cr [13,14,15,16]. These elements are reported to have some level of toxicity and are not desired to be used as a component of the implants’ material.

Commercially pure Ti has a Young’s modulus of 100–110 GPa, which is higher than that of cortical bone (20–25 GPa), which, in turn, causes the stress shielding effect and results in bone resorption [17]. Titanium alloying with β-stabilizer elements allows the production of a low modulus material which is desirable in the medical field [18]. The formation of the β-type structure leads to the reduction of mechanical mismatch between the elasticity of bone and implant. Specific β-stabilizers, such as Ta, Nb, Zr, and Hf, for titanium alloys further improve the material’s properties [19,20,21,22,23]. Non-toxic and non-allergenic Nb represents a relatively new and promising implantable biomaterial, and has been proved to be biocompatible in vitro and in vivo [2]. Titanium alloys that contain 40–45 wt.% Nb have an elastic modulus of about 60 GPa, compared to more than 110 GPa of Ti-6Al-4V, which is the most-used alloy in implant manufacturing [24].

Another approach for the elastic modulus of cortical bone is the introduction of a controllable level of porosity [25]. According to I. H. Oh et al. [26], it is possible to reduce the elastic modulus of material by introducing pores. Moreover, the presence of porous structures promotes bone ingrowth that could improve the osseointegration of the implant [27]. To achieve a high level of osseointegration, implants should mimic the morphology, structure, and properties of bone tissue [25,28,29,30]. The interconnection and size of the pore structure are important factors affecting bone ingrowth. Large pores (100–150 µm and 150–200 μm) lead to significant bone ingrowth, whereas smaller pores (75–100 μm) lead to the ingrowth of demineralized tissue. It is reported that only fibrous tissues penetrate pores of 10–45 μm and 45–75 μm in diameter. It could be concluded that the optimal pore size for metallic scaffolds should be in the range of 100–400 μm according to S.F. Hulbert et al. [31].

Furthermore, the medical application of 3D printing has led to growing demand for personalized implants. Three-dimensional printing (also referred to as additive manufacturing, (AM)) has become widespread for the manufacturing of anatomical models, customized implants, and surgery planning [25,29]. Metallic personalized implants are of particular interest to researchers and industry due to the opportunity for significant customization of the product. Laser powder bed fusion (LPBF), a type of AM method, provides the possibility to produce porous metallic structures of unique shapes and sizes. There are several publications devoted to the production of porous metallic scaffolds and structures using an LPBF method, in particular, and AM, in general [32,33,34,35,36]. In a 2017 review, H. Shahali et al. [25] describe the LPBF of Ti-6Al-4V porous structures and scaffolds. It was noted that the ideal pore size ranges from 250 to 450 μm. The porosity of produced samples was in the range of 80–95% with a Young’s modulus of 0.12–1.25 GPa. In addition, the researchers confirmed that the pore size of titanium implants manufactured by LPBF has a considerable influence on bone ingrowth. The porous structure was introduced through non-stochastic cellular structure design, where structural units, such as polyhedral, cubic, truncated octahedron, or rhombic dodecahedron, were arranged periodically to achieve a porous architecture [32]. Hence, the unit shape of LPBF scaffolds has become a key factor for the controllable manufacturing of porous design [37,38,39,40,41,42,43]. Zhang et al. [12] carried out experiments and numerical simulations with the aim to form samples made of Ti-6Al-4V alloy with a significant fraction of porosity and estimated their physical and mechanical properties. The porosity of as-built samples was in the range of 40–55% and the simulated porosity was 38–75%. The authors noted the relationship between the porosity and apparent elastic modulus. The experimentally determined Young’s moduli of samples with different porosity levels were in the range of 3.3–5.7 GPa. Similarly, D. Pattanayak et al. [44] highlighted the relationship of porosity to the compressive strength of the LPBF samples made of pure titanium gas-atomized powder. They showed that the porosity of 75–55% could affect the values of compressive strength in the range of 35–120 MPa. In the paper by I. Polozov et al. [45], samples for applications in the aerospace industry, made of Ti-5Al, Ti-6Al-7Nb, and Ti-22Al-25Nb alloys with a corresponding porosity of 1%, 0.6%, and 2.7%, respectively, were studied. The mechanical properties of these alloys were comparable to the commercially available materials of the same composition.

For the use in AM, however, a powder material should have certain parameters, such as shape and size [46]. The powder material is commercially produced by spheroidization methods, which include centrifugal gas atomization and plasma spraying [47,48]. An alternative to the spheroidization method that is recently gaining more attention is mechanical alloying [49,50]. The mechanical alloying allows production of powders of a size suitable for AM with a nanosized structure that provides extraordinary properties to the finished product. During the mechanical alloying process, powder particles receive enough energy for phase transformations and the formation of solid solutions. In our previous papers, we described the influence of milling time and process atmosphere on structure, particles’ morphology, particles’ size distribution, phase, and elemental compositions of resulting powder using a planetary ball mill [51,52,53,54]. According to the obtained results, we found an optimal mode of mechanical alloying for powders of Ti and Nb.

It should be noted that the powder material for Ti-Nb alloy production is not commercially available, or can be provided by the companies dealing with AM only in large quantities, which are not required for the purpose of science and research. The spheroidization methods of powders for AM are expensive and provide a small yield of applicable material. Methods of mechanical alloying, however, could be an effective cost-efficient alternative to spheroidization [50].

Finally, significant global effort has been made by researchers to produce a variety of AM materials, with different designs and properties, for biomedical applications. Furthermore, it should be noted that only a few papers have reported attempts to produce porous or dense low-modulus model implants from Ti-Nb alloys using AM, or any other production method, such as powder metallurgy or casting [55,56,57,58,59]. The aim of this study was to contribute to the field of AM by reporting on manufacturing scaffolds made of hypoallergenic Ti-Nb alloy, which are needed commercially in the form of dense and porous design samples. We describe the manufacturing route of Ti-(40–45) wt.% Nb powders using the method of mechanical alloying followed by the thorough research of LPBF manufacturing regimes for both dense and porous samples, their structure, and properties. Biocompatibility tests were also carried out for manufactured samples to test the osteoblast-like cell line (MG-63) response to the produced surfaces as the first step towards further application in the biomedical field.

## 2. Materials and Methods

### 2.1. Ti-Nb Alloy Powder Production

**Figure 1:**
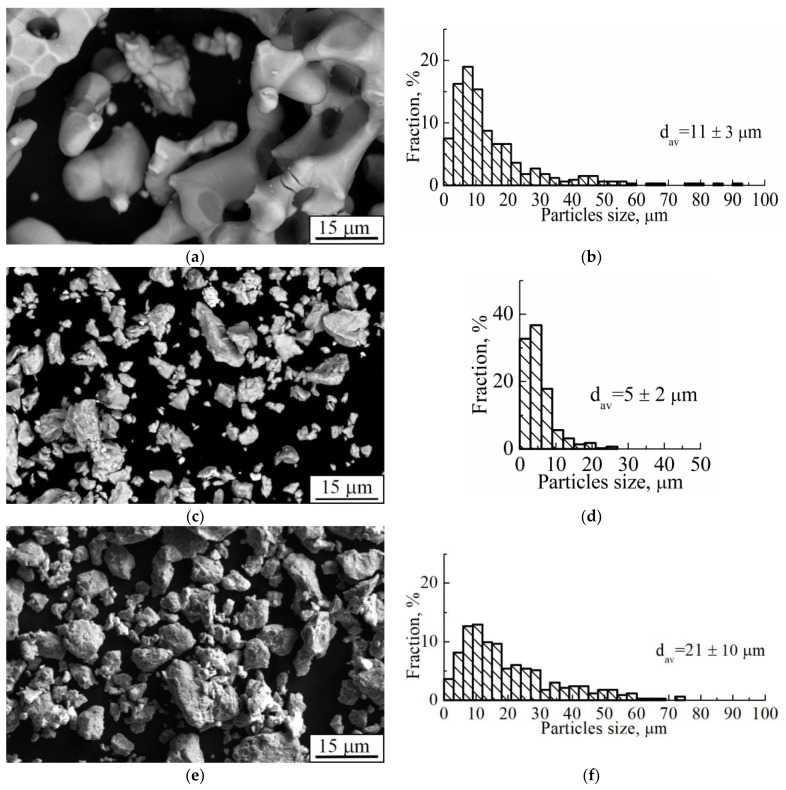
(**a**,**c**,**e**) SEM-image and (**b**,**d**,**f**) size distribution of powder particles of (**a**,**b**) titanium, (**c**,**d**) niobium, and (**e**,**f**) Ti-(40–45) wt.% Nb after mechanical alloying (d_av._ stands for the average particle size).

Pure titanium (Ti) and niobium (Nb) powders were manufactured by calcium hydride and hydrogen reduction, respectively (Polema, Tula, Russia). The average size of Ti and Nb particles was 10 μm and 5 μm, respectively (Figure 1a–d). The pure powders were mechanically alloyed in an AGO-2C planetary ball mill [51]. The mechanical alloying of Ti-Nb powder was carried out in Polzunov Altai State Technical University. According to [60], solid solution formation in this mill can be performed at much smaller periods when compared to other well-known and commercially used mills. Alloying was carried out in an argon atmosphere for 15 min. The particle size distribution of the resulting Ti-Nb powder was in the range of 5–75 μm (Figure 1e,f).

### 2.2. LPBF of Ti-Nb Alloy

**Figure 2:**
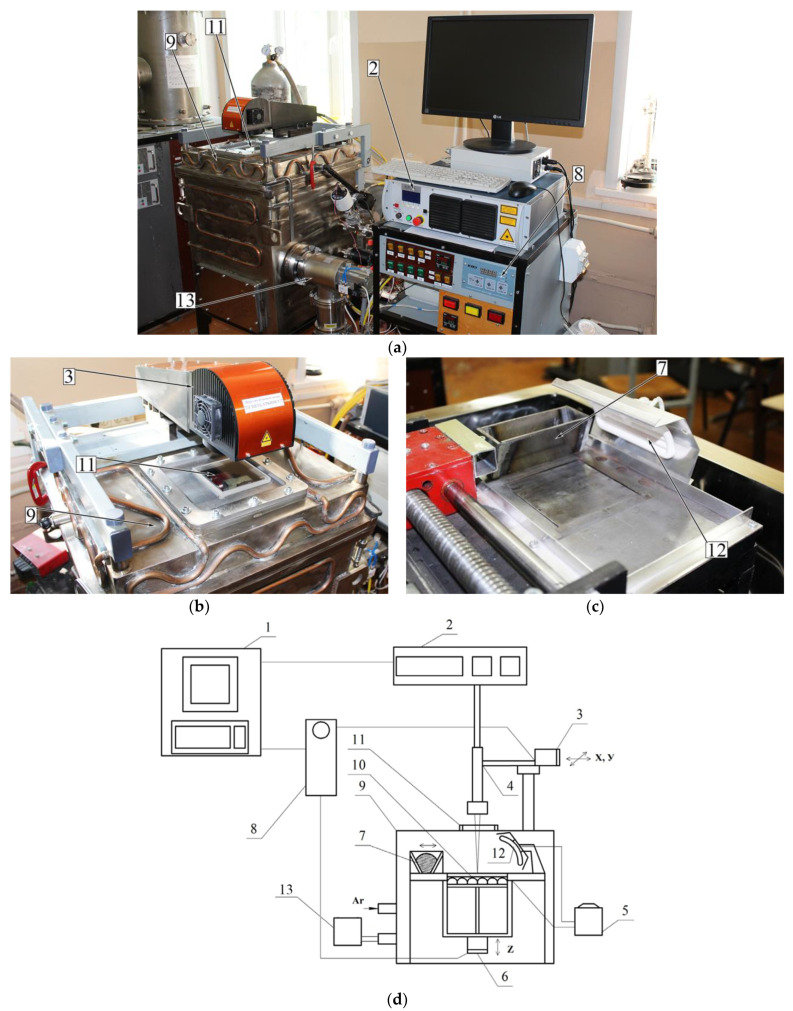
VARISKAF-100 MVS machine: (**a**) overall view, (**b**) scanning system, (**c**) feeder, (**d**) schematic view; 1—computer; 2—laser; 3—system providing X-Y laser displacement; 4—collimator; 5—substrate temperature control system; 6—system providing displacement of the baseplate along Z axis; 7—feeder; 8—CNC; 9—vacuum chamber; 10—spiral heater; 11—chamber window; 12—infrared heater; 13—vacuum pump.

For the bulk sample production, the previously manufactured powder of Ti-(40–45) wt.% Nb alloy was used in a custom-made VARISKAF-100MVS laser fusion system (Figure 2). This is equipped with an ytterbium laser with a 1070 nm wavelength and a vacuum chamber, which can be filled with inert gases (argon, nitrogen, etc.). In our case, the protective atmosphere was Ar with the chamber base pressure of 10^–3^ Pa. In the present paper, we study porous and dense samples produced with different melting modes. The baseplate and powder’s surface were preheated to 573 K in both cases.

#### 2.2.1. LPBF of Ti-Nb Porous Samples

For porous sample manufacturing, a single layer sintering with subsequent remelting was performed. The detailed scheme can be found in the Supplementary Material.

The remelting–sintering strategy included the formation of each layer of the sample comprised of two stages: sintering and remelting. At the sintering stage, following the predefined strategy, a contour of a sintering zone was formed by a laser beam. Inside the formed contour a powder was sintered along the straight lines (tracks) with a hatch distance of 0.4 mm. The same laser strategy was repeated with a 0.2 mm step for the already sintered tracks. The same sintering strategy was repeated along the normal to the sintered tracks direction. After the sintering stage in both directions was performed, the layer was formed. To perform melting of any residual powder particles into a liquid state and ensure the complete sintering, the remelting stage was performed. Moreover, the remelting stage allowed us to reduce the level of internal stress. This stage was performed using a chess-like strategy for even and odd layers. This sintering strategy is based on our previous experience reported elsewhere [61]. From this experience, we discovered that the complex strategy of beam movement allows a more homogeneous heat dissipation that leads to a decrease in local hot metal coagulation and, overall, reduces the residual stress. During the melting stage, a 50 µm powder layer was sintered with a scanning speed of 0.06 m/s, 80 W of power, a laser beam focal spot of 100 μm, and a hatch distance of 0.4 mm. During the remelting stage, the laser beam power was increased to 100 W and the laser beam scanning speed was varied for even and uneven layers (0.1 and 0.06 m/s, respectively), the scanning step was set to 0.05 mm, and the laser beam focal spot was increased to 150 μm.

#### 2.2.2. LPBF of Ti-Nb Dense Samples

**Figure 3:**
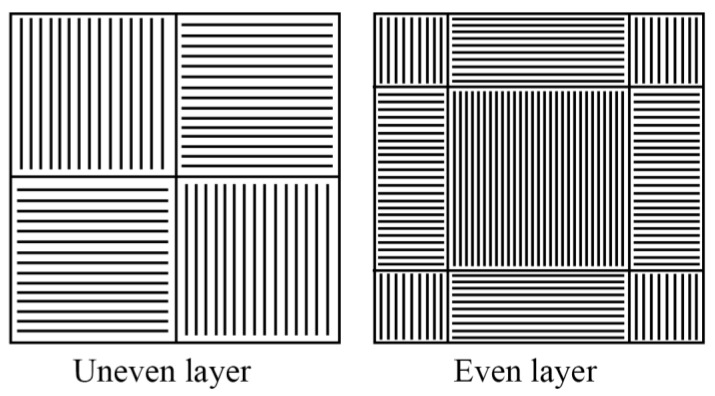
Scanning strategy of the dense LPBF samples.

For the dense sample manufacturing, the scanning strategy was changed. To reduce the porosity, it was required to provide a higher level of internal energy within the fused powder. An increased level of internal energy would prevent balling effects and disordered liquid solidification [25]. For this purpose, a repetitively-pulsed mode of laser beam scanning also called “modulation mode” was applied. For dense samples, an “area cross-hatch” mode was used with the following parameters: laser beam scanning speed—0.07 m/s; power—100 W, frequency of impulse modulation—300 Hz, laser beam focal spot—100 μm, scanning step—100 μm. The thickness of the powder layer was 50 μm. The scanning strategy is represented in Figure 3.

### 2.3. Microstructural Characterization of LPBF Ti-Nb Samples

The surface morphology, in addition to the macro- and microstructure of the produced samples, and their elemental and phase compositions, were studied by optical metallography (OM), scanning electron microscopy (SEM), energy dispersive spectroscopy (EDS), X-ray fluorescent analysis (XRF), transmission electron microscopy (TEM), X-ray diffraction (XRD) analysis, and X-ray computed tomography (CT). OM, SEM, EDS, TEM and XRD studies were carried out using the equipment of “Nanotech” Common Use Center of ISPMS SB RAS. XRF and part of SEM results were obtained using the equipment of Tomsk Material Science Common Use Center.

OM and subsequent SEM studies of LPBF samples were carried out using Carl Zeiss Axio Observer A1m optical microscope (Zeiss, Jena, Germany) and SEM-515 (Philips, Philips, Netherlands) equipped with an EDS Genesis (AMETEK, Berwyn, PA, USA), respectively. Before observation, the surface of LPBF samples were mechanically grinded with P400, P600, P1000, P1500 sandpapers and polished with 14/10 and 1/0 polishing paste. Then, the samples were electrolytically polished in a mixture of sulfuric, hydrofluoric, and nitric acids in a 6:1:3 volume ratio.

XRF of powder material was carried out with XRF-1800 (Shimadzu, Nakagyo, Japan). A voltage of 40 kV and a current of 95 mA were applied.

The microstructure of manufactured powders was analyzed with TEM JEM-2100 (JEOL, Akishima, Japan) equipped with an Inca EDS system (Oxford Instruments, Abingdon, UK). The preparation of samples for TEM analysis was performed using an Ion Slicer EM-09100 IS (JEOL, Akishima, Japan). For the TEM studies, thin foils were prepared. For this purpose, LPBF samples were cut with a thickness of 300 µm. Regarding the powder material, it was submerged in epoxy for further processing. The TEM samples were further thinned to 100–150 µm by grinding. Obtained samples were further thinned by ion beam etching in an Ion Slicer. The applied acceleration voltage was 6 kV and the descent angle was 1.5–3°, depending on the studied area. TEM studies were carried out at 100–800× magnifications and an accelerating voltage of 200 kV. Modes of light- and dark-field, and selected area electron diffraction were applied.

X-ray diffraction analysis was carried out with a DRON-7 (Bourevestnik, St. Petersburg, Russia) diffractometer with CoKα radiation. The 2θ angle range was set as 10–100°, and the scanning step was 0.02° with an acquisition time of 3 s. Phase identification was carried out according to #00-044-1294, 00-034-0370, 01-071-9957, 00-017-0102, and 01-071-9955 PDF-cards taken from the International Center for Diffraction Data PDF4+ database. 

The X-ray computed tomography (CT) measurements were performed on a GE v|tome|x L 300 micro-CT scanner (GE Sensing & Inspection, Wunstorf, Germany). Only porous samples were measured. The tube voltage and current were set to 100 kV and 90 µA, respectively. A voxel size of 3.33 µm^3^ was achieved. VG Studio MAX version 3.2.1 (Volume Graphics GmbH, Heidelberg, Germany) and Fiji ImageJ (Rasband, W.S., U. S. National Institutes of Health, Bethesda, Maryland, USA) software were used for inclusions and porosity analysis. The 3D visualization was performed in Avizo (Thermo Fisher Scientific, Waltham, MA, USA) software.

### 2.4. Cytotoxicity Study of as Manufactured LPBF Samples In Vitro

The cytotoxicity of LPBF samples was investigated in vitro using a human osteosarcoma cell line (MG63) that was purchased from ATCC (CRL-1427, Manassas, VA, USA). For cell culture experiments, Dulbecco’s Modified Eagle’s Medium (DMEM), fetal bovine serum (FBS), penicillin-streptomycin, and L-glutamine solution were obtained from Sigma-Aldrich (Sigma-Aldrich, Darmstadt, Germany). 3-(4,5-dimethylthiazol-2-yl)-2,5-diphenyltetrazolium bromide (MTT) were purchased from Thermo Fisher.

MG63 was cultured in DMEM containing 10% FBS and penicillin–streptomycin mixture in a humidified incubator at 37 °C under 5% CO_2_ atmosphere. The MG63 were passaged upon 80−90% confluency.

An MTT assay was used to evaluate the cell viability of MG63 cell line. The cells were seeded directly onto the samples’ surface using the direct seeding method. A total of 40,000 cells were seeded in 60 µL of medium, and on dense and porous LPBF samples and cell culture plastic in 12 well plates. After two hours of incubation in order to allow cells to attach to the surface of a material, the culture wells were filled with a warm (37 °C) medium. After three and seven days, the medium was removed, and cells were washed twice with sterile PBS before the addition of 120 µL of MTT reagents to each culture well for four hours. The liquid was aspirated, and the formazan precipitates were dissolved in DMSO/ethanol solution. The optical densities were measured at 550 nm using a microplate spectrophotometer (POLARstar Omega, BMG LABTECH, Ortenberg, Germany). The cells on a cell culture plastic were used as the control group. Three separate experiments with four samples per each type were carried out.

The separated groups of samples were stained using a LIVE/DEAD kit with Calcein AM (17783 Sigma-Aldrich, Darmstadt, Germany) and Propidium iodide (P4864 Sigma-Aldrich, Austria) in concentrations of 5 μmoll and 1.5 μmoll, respectively. The LPBF porous and dense samples were stained after three and seven days of culture. Fluorescence was visualized using a fluorescence microscope (Axio Imager.A1, Zeiss, Jena, Germany). Potentially viable and damaged cells were distinguished because it was assumed that the viable cells appeared green, whereas non-viable or membrane compromised cells appeared red. All data is expressed as mean ± SD of three independent experiments. The *p* values < 0.05 were considered statistically significant.

## 3. Results and Discussion

**Figure 4:**
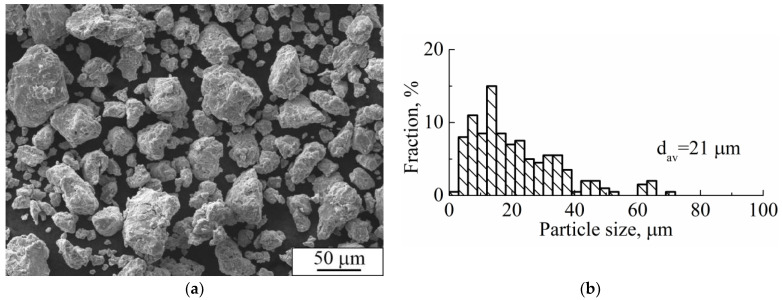

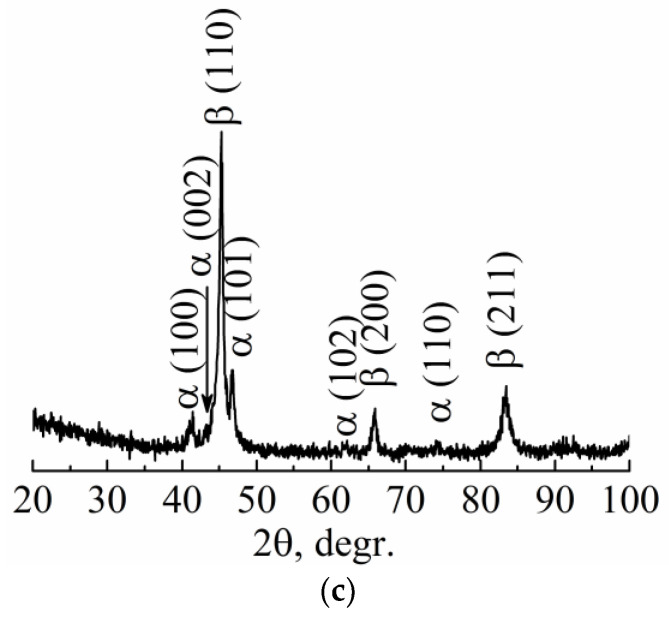
(**a**) SEM image, (**b**) particle size distribution, and (**c**) XRD pattern of mechanically alloyed Ti-Nb powder (d_av._ is the average particle size).

**Figure 5:**
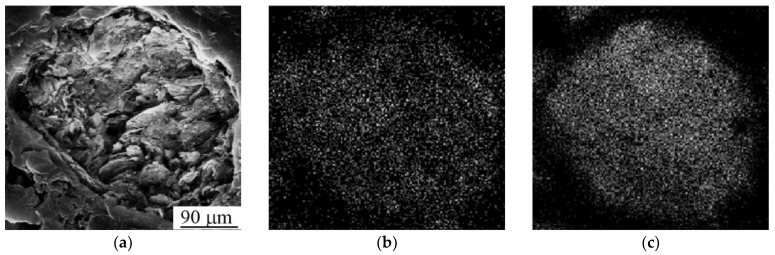
(**a**) SEM image and mapping patterns of (**b**) Ti and (**c**) Nb.

The SEM image of Ti-Nb powder particles in the form of elliptical-like agglomerates after mechanical alloying is represented in Figure 4a. The particle size distribution is represented in Figure 4b. The average particle size was 21 μm. A phase composition is represented by the main β- and the second α-phase. The fraction of the α-phase estimated from XRD data was 32 vol.%. According to XRD data, the calculated lattice parameter of the β-phase was a = 0.3292 ± 0.0003 nm, which corresponds to the value of a parameter for the phase of Ti-Nb alloy. This phase represents a solid solution of Ti and Nb based on the bcc lattice of Nb or the bcc high-temperature phase of β-titanium [62]. SEM and EDS studies have shown the uniform distribution of Ti and Nb in the particles (Figure 5) [63]. The concentration of Nb was found to be 44 ± 5 wt.%. according to EDS. The XRF measurements showed an absence of Fe particles in the powder, which is of importance because of the Fe toxic effect [32]. It is known [45,64] that mechanical alloying or ball milling usually leads to powder contamination with Fe particles. This is usually associated with a prolonged milling time (up to 40 h), which results in erosion of milling vials or milling balls. In our case, we obtained impurity-free powder because we used only 15 min of treatment with constant water cooling, compared to 12 h [45] or 40 h [64] reported in the literature. In our case, we conclude that the mechanical alloying for 15 min led to the formation of impurity-free Ti-Nb alloy. Similar results were obtained by authors in [65].

According to the phase diagrams of the Ti-Nb system and our previous reports [51,52,53,63], a formation of solid solutions based on the bcc lattice during the process of mechanical alloying can occur in this system by two simultaneous non-competitive mechanisms. According to the first mechanism, which is based on an α-Ti lattice, Nb-atoms are introduced into the α-hcp lattice up to the concentration of 5 wt.%. Thereafter, due to the non-equilibrium high-energy processes occurring during the mechanical alloying, the upper limit of Nb solubility in the α-Ti lattice is extended up to 40 wt.%. Such a high concentration of Nb atoms in the α-Ti lattice leads to the reverse polymorphic transformation and formation of the β-bcc phase based on the β-Ti lattice, which is a β-TiNb solid solution. The second mechanism suggests an involvement of the β-Nb lattice. The Nb lattice has an upper limit of Ti atoms solubility of 60 wt.%. Therefore, due to the mechanical alloying, β-NbTi is produced, which is a solid solution of Ti and Nb based on the Nb-bcc lattice. In addition, it should be noted that if the milling time was insufficient for the full phase transformation, there would be a mixture of more than two phases, which could be α-Ti, α-TiNb, β-TiNb, β-NbTi, and β-Nb.

According to this description, as a result of the mechanical alloying of 55 wt.% Ti and 45 wt.% Nb, a mixture of two solid solutions could be produced: β-TiNb and β-NbTi. Both of these share the same type of lattice (bcc), the same values of lattice parameter (a = 0.329 nm), and the same XRD patterns. However, these phases can be distinguished from each other based on the prevalence of an element, which resembles the basis lattice of the alloy. It is of vital importance to distinguish the phase composition, because pure β-Nb and pure β-Ti have a large difference in their physical properties (such as density, melting temperature, heat capacity, and heat conductivity). Therefore, it is obvious that the β-TiNb and β-NbTi solid solutions also possess significantly different properties.

**Figure 6:**
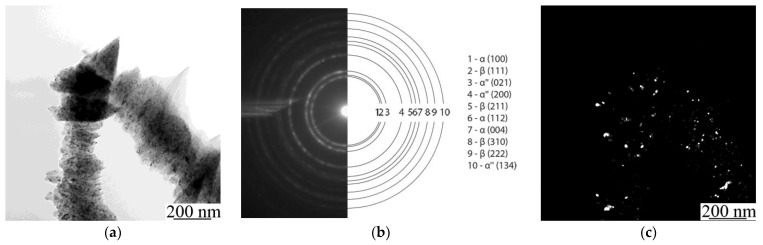

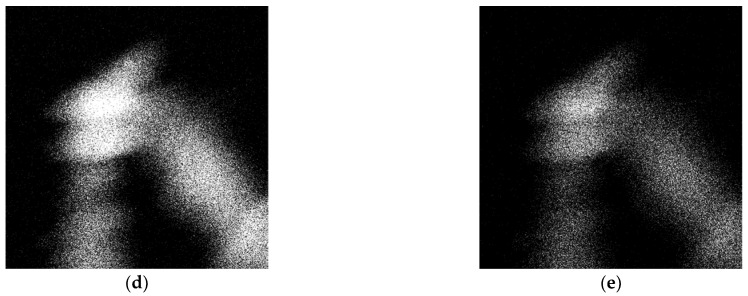
(**a**) TEM light-field image, (**b**) SAED pattern, (**c**) dark-field image in combined α(100) and β(111) reflex and maps of elements’ distribution: (**d**) titanium and (**e**) niobium.

To estimate the concentration and distribution of Ti and Nb in the alloy and to prove whether a solid solution of Ti-Nb was formed in the powder, a detailed analysis of a thin Ti-Nb structure using TEM with an in-column EDS was carried out (Figure 6). As can be seen in Figure 6, powder agglomerates with sizes of tens of µm (Figure 6a), that were formed due to mechanical alloying, consist of nanosized grains with an average size up to 90 nm, which can be seen in a dark-field image (Figure 6c). Thus, the powder particles have a nanoscale structure, which could affect the process of LPBF and provide the specific structure and properties to the manufactured material. According to selected area electron diffraction (SAED), the phase composition of the studied powder is represented by α-, β-, and α″-phases (Figure 6b). The latter is the non-equilibrium phase, which is usually formed after quenching or a plastic deformation [62]. Because we did not observe any traces of the α″-phase in the XRD patterns, it can be concluded that there was a small amount of this phase in the powder, which cannot be identified by an XRD method (Figure 4c). As can be seen in Figure 6b, the ring-type reflexes from the α″-phase are broadened. This indicates that the α″-phase is represented by the structural elements of small size. As was previously shown (Figure 4c), the XRD pattern has a noisy background. Both of these facts highlight that the α″-phase is represented by the structural elements of nanoscale size. In addition, the presence of this phase confirms the fact that the phase transformation during the mechanical alloying occurs, and solid solutions of Ti-Nb were formed. The results of an EDS in column mapping of elements are represented in Figure 6 d,e. The obtained data are in the agreement with the previously gathered results obtained during SEM elemental mapping from the surface of the particles. TEM and SEM-EDS confirmed that the Ti and Nb are uniformly distributed, not only in the powder particles of micro scale size, but also throughout the nanostructured powder particles. The obtained data with previously shown results of XRD, XRF, and SEM highlighted the formation of a mixture of two Ti-Nb solid solutions, containing α- and β-phases, in the produced nanostructured powder. Mechanical alloying provides a uniform mixture of initial components (Ti and Nb) in the resultant powder with a nanoscale structure, which creates a required condition for the partial β-phase formation, which in turn forms powder agglomerates consisting of nano-sized grains. The obtained nanostructured powder material was used to manufacture dense and porous types of LPBF samples.

**Figure 7:**
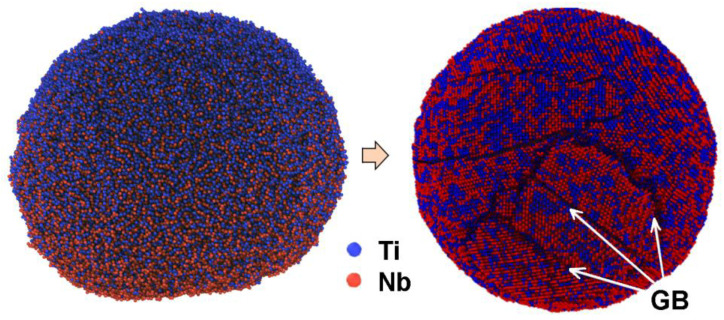
Initial and resulting structure of Ti-Nb solid solution obtained after MD simulation of the Ti and Nb particles’ solution solidification process.

Before carrying out the LPBF of Ti-Nb samples, the numerical simulation of the mixture crystallization that consists of pure Ti and Nb nanoparticles during their fusion was completed, following the framework of the molecular dynamics method (MD) [66]. The description of the applied numerical model, its validation, and verification was reported elsewhere [67]. According to the results of the simulation, the value of the temperature gradient imitating the process of the laser beam influence during the LPBF affects the fraction of atoms that form on the bcc structure of the Ti-Nb alloy. It was found that, with an increase in the crystallization duration, the fraction of atoms forming the β-phase increases nonlinearly. The upper limit of β-phase presence was found to be 85 vol. %. The remaining 15 vol. % is attributed to the Ti and Nb atoms that are predominantly situated along the internal boundaries of the formed nanograins (GB). The typical view of a Ti-Nb solid solution structure that was produced during the direct simulation by the MD method of the crystallization process after the fusion of pure Ti and Nb particles solution is represented in Figure 7. The applied numerical model showed that, with the increase in crystallization rate, the volume fraction of atoms forming the β-phase decreases. For example, the 57 vol. % of atoms attributed to bcc- was found for the middle rate of crystallization and only 11 vol. % was found for the high rate of crystallization. This result was confirmed by the experimental studies. It is known that, with the increase in the crystallization rate of Ti-Nb alloys, the structure is characterized by the formation of non-equilibrium phases with other types of lattice [55,56,58].

**Figure 8:**
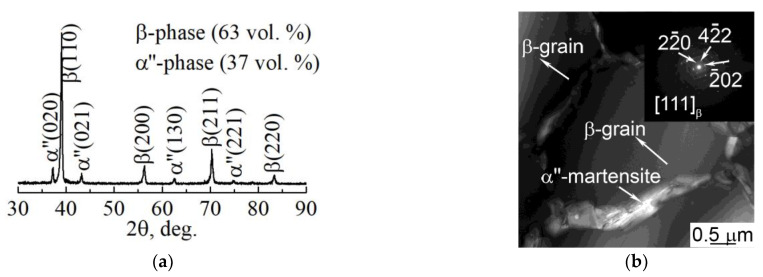
(**a**) XRD pattern and (**b**) TEM light-field with corresponding SAED pattern of LPBF porous sample.

For a microstructural material analysis of both dense and porous LPBF samples, TEM was used. It was established that in both types of samples the uniaxial grains with nanosized precipitations inside the grain and throughout the grains’ boundaries are visible in TEM (Figure 8b). As previously shown in various reports [61,67,68], these are the main β-phase grains with the size of tens of µm and precipitations of the non-equilibrium α″-phase with the size of dozens of nm. The length of the α″-phase’s grains does not exceed 1000 nm and that transverse size is in the range of 90–200 nm. According to the EDS results, α″-phase nanoscale grains contain a reduced amount of Nb that does not exceed 20 wt.% in comparison to the β-phase grains. Such elemental composition is typical for the nanoscale martensite α″-phase. The XRD and TEM analyses (Figure 8) reveal the phase composition, which is represented by 63 vol. % of the β-phase and 37 vol. % of the α″-phase (Figure 8a). This indicates that 63 vol. % of atoms that are forming the sample are formed in the bcc lattice, corresponding to the β-phase. As was shown previously, in the results of numerical simulation, such a volume fraction of bcc atoms corresponds to the middle rate of crystallization. Thus, the results of numerical simulation and experimental results are in good agreement. It is worth noting that the grains that correspond to the β-phase are significantly larger than the grains corresponding to the α″-phase (Figure 8b).

**Figure 9:**
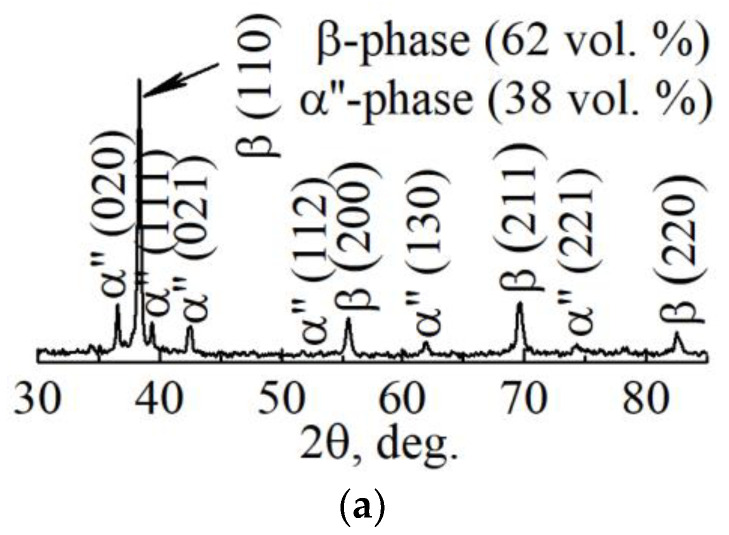

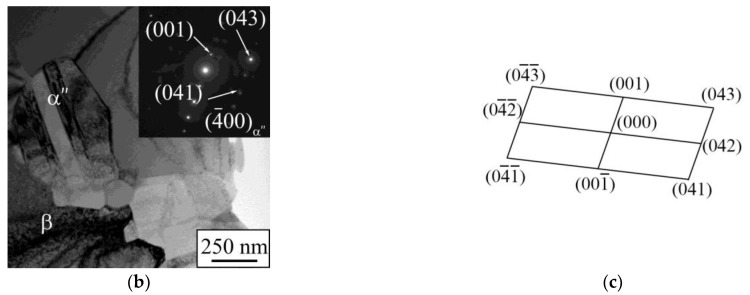
(**a**) XRD pattern, (**b**) light-field TEM-image with corresponding SAED pattern, and (**c**) phase identification scheme of LPBF dense sample.

The results of XRD and TEM studies of the structure of the dense sample are represented in Figure 9. The phase composition of the dense sample is practically identical to the composition of the porous one (Figure 9a). The quantities of the main β-phase in dense and porous samples were 62 vol. % and 38 vol. % for the α″-phase. Thus, according to the volume fraction of the bcc β-phase, the crystallization rate of the dense sample also corresponds to the middle rate of crystallization.

According to the TEM images (Figure 9b,c), it is obvious that an α″-phase is represented by the grains, which are significantly smaller than those of the β-phase. Thin plates of the α″-phase have a length not exceeding 700 nm and a transverse size that lies below 100 nm. The Nb concentration within the nano-scaled α″-grains does not exceed 25 wt.%, which is the same as for the porous LPBF sample. TEM results confirm that the nanoscale α″-phase precipitations are localized inside microscale β-phase grains and throughout their boundaries (Figure 8b and Figure 9b) [67,68]. The presence of the nonequilibrium nanoscale α″-phase is caused by rapid cooling conditions of samples during the LPBF process and elemental composition’s non-uniformity [67,68]. In addition, this could be due to the presence of a small amount of nanoscale α″-phase in the initial mechanically alloyed powder applied for LPBF. Such structure features and phase composition provide a reduced Young’s modulus of the alloy (in comparison with the modulus of pure components), which is in the range of 60–85 GPa for porous and dense samples, and increased microhardness of 7500 MPa [67,69].

**Figure 10:**
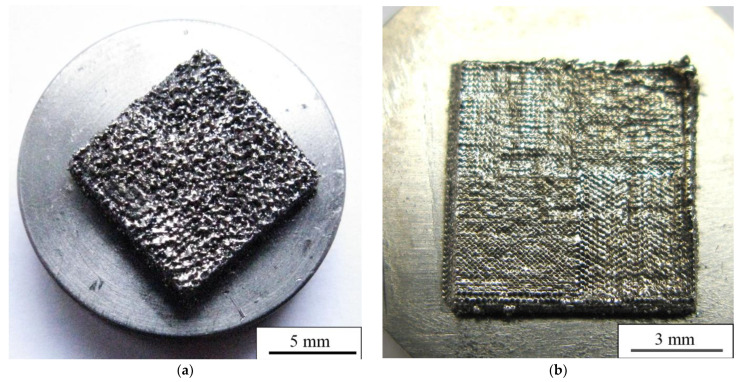
Top view of (**a**) porous and (**b**) dense LPBF-sample surface.

Photographs of a top view of the bulk porous and dense LPBF samples are shown in Figure 10a,b, respectively. The surface with visible open porosity is typical for porous LPBF samples [54]. The formation of an open porosity and surface roughness can be explained as follows. As described in the Materials and Methods section, the molten pools are layered under each other during the sintering as a result of the laser beam movement, which leads to the formation of an uneven surface profile after its rapid crystallization [54]. The droplets that were created by the laser beam appear on the surface during the melting stage, to be later precipitated on the consolidated surfaces of molten pools. Then, the complete crystallization and formation of the rough surface with visible cavities appears, which is depicted in Figure 10a [69]. In Figure 10b, a more uniform and much smoother surface of a dense sample is represented. In the top view, the boundaries between sintering areas and traces of tracks are visible. It has been well reported that the value of roughness is an important parameter for biological applications. A certain type of morphology and parameters of micro-roughness influence cell response and guide cell-specific behavior [2,25,29,70,71,72,73,74,75,76,77]. As described in [29], surface morphology of the top layer does not depend on the melting mode, because it is affected by the powder particle morphology. In our case, we observed the opposite. Surface morphology in our samples depends on scanning strategy and LPBF mode. In addition, authors of [29] show that droplets of a powder melt occur during LPBF, which provides the surface roughness required for cell adhesion. In our case, both porous and dense samples have the surface and the value of roughness required for good cell adhesion.

**Figure 11:**
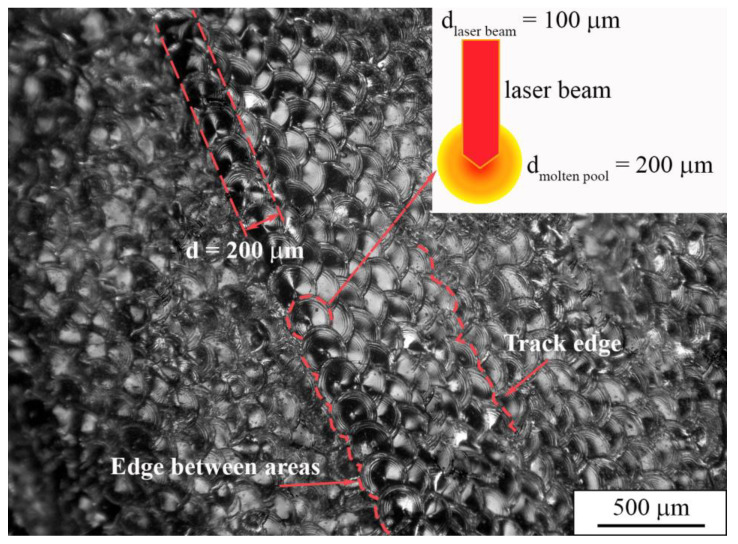
Multifocal digital image of the top surface of a dense sample.

For a more detailed analysis of the morphology of the sample surface, multifocal digital imaging was employed. As can be seen in Figure 11, the traces of laser tracks and edges between fusion areas reflect a predefined scanning strategy of a laser beam. The boundaries of molten pools are clearly defined. Due to the heat dissipation during the laser modulations in the LPBF process, a molten pool diameter appears to be two-fold larger than the laser beam focal spot. We assume that the shape of molten pools that is represented in Figure 11 is connected to the laser modulation mode that was used during the LPBF manufacturing of a dense sample.

**Figure 12:**
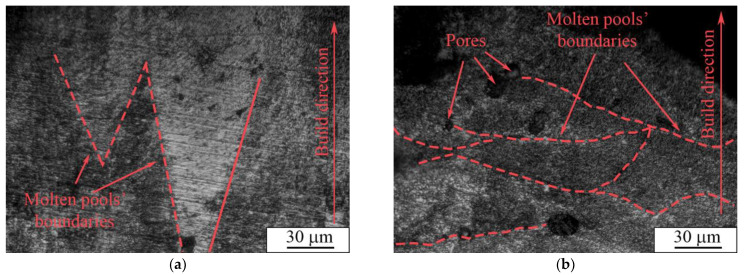
Optical images of (**a**) dense and (**b**) porous samples’ transverse sections.

In the paper by J. Yang et al. [78], AM parameters concerning the molten pool shape and structure were thoroughly studied. The molten pool diameter described in the report was in the range of 200–250 µm, which is close to that in our case (200 μm). However, the energy input described in the paper ranged from 2 to 1 J/mm, whereas in our case it was in the range of 1.3 to 1.4 J/mm for porous and dense samples, respectively. Furthermore, the authors show a “tear-drop” shape of a molten pool in a cross-sectional view, whereas in our case we detected a V-shaped type, as can be seen in Figure 12a. Moreover, the height of the molten pools, which varied between 70 and 200 µm, was larger than the thickness of a single powder layer used for the LPBF process, which was set to be 50 µm. The difference in shape and thickness of the molten pool can be ascribed to the remelting stage with a laser modulation mode, which affected the heat dissipation direction. As reported in [78], the V-shaped molten pools provide better refusion between formed layers and allows dense samples to be produced, whereas the U-shaped pools create conditions for open and isolated porosity formation.

A cross-section image of a porous sample shows the arch-shaped molten pools’ boundaries (Figure 12b), contrary to the U-shape presented in [78]. An arch-shape is frequently reported for LPBF samples [79,80]. This shape can appear due to heat dissipation towards the sample’s edges. As described in [80], the molten pool arch-shape is related to concurrent laser movement, i.e., scanning strategy significantly affects the molten pool shape. This heat distribution due to the LPBF mode for porous samples cannot provide dense fixation between certain parts of powder layers, which leads to the formation of open porosity.

**Figure 13:**
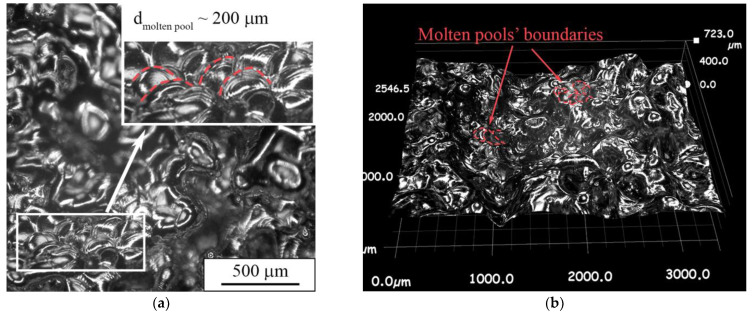
Multifocal digital image of a porous sample’s top surface in (**a**) 2D and (**b**) 3D mode.

As can be seen in the confocal image of the top surface of the porous sample, round-shaped molten pools are observed in certain areas on the surface (Figure 13a). In the 3D image of the top surface, it can be seen that the molten pools have an irregular shape (Figure 13b). The shape changes from a round type in the peak point to a “tear-drop” shape in lower areas, and even to irregular shapes. The observed type of surface morphology could be connected to the remelting stage, during which the free heat dissipation is realized. This is implicitly confirmed by appearance of molten pools along the building direction (Figure 12b). An arch-shaped molten pools’ boundary indicates the strong heat dissipation towards the sample’s edges and a weak dissipation towards the substrate. Thus, we conclude that the LPBF mode of a single layer’s sintering with subsequent remelting using a “cross-hatch” strategy leads to “all-round” heat dissipation and partial dense fixation of layers with simultaneous formation of porosity.

**Figure 14:**
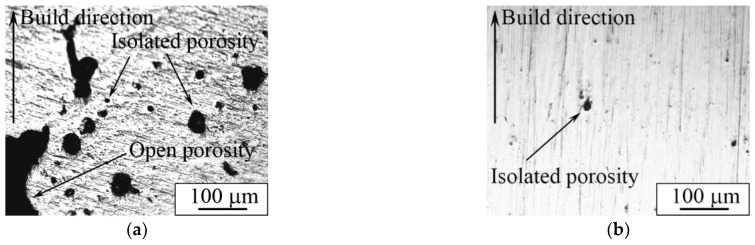
Optical image of (**a**) porous and (**b**) dense LPBF samples’ transverse section.

An optical micrograph of the cross-section of porous and dense samples is represented in Figure 14. An isolated porosity in the porous sample was estimated according to the secant method [81] and was equal to 3.6 ± 0.5 vol. % (Figure 14a). The presence of porosity in our case is desirable, because this material is planned to be used for implantable devices. However, the necessity of an isolated or open porosity to improve osteointegration remains a topic of debate. According to the reported data, an open porosity promotes and intensifies bone cell proliferation into the implant [17], whereas isolated porosity provides decreased values of apparent Young’s modulus [26]. In addition, it has been suggested that AM samples with a simple grid-like structure and relatively large isolated porosity could be successfully used as a drug carrier [82].

**Figure 15:**
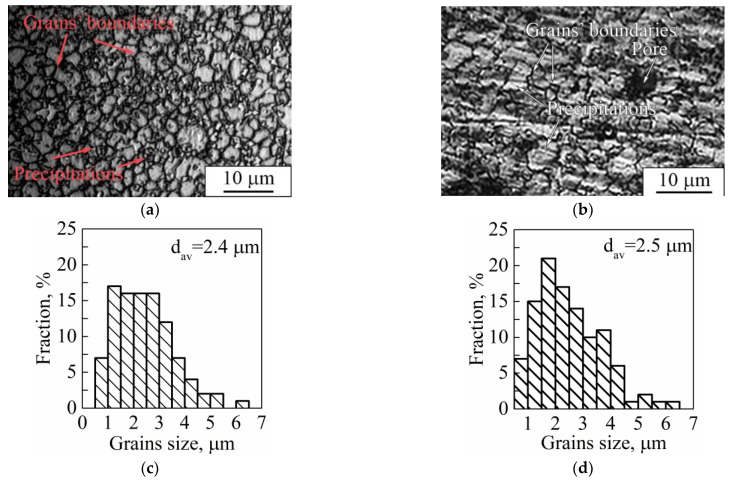
Optical images of (**a**,**b**) microstructure and (**c**,**d**) grain size distribution of (**a**,**c**) dense and (**b**,**d**) porous samples (d_av_ is the average grain size).

Carrying out a comparison between grain sizes of dense and porous samples, one can observe that grains are insignificantly larger in the dense sample than those in the porous samples (Figure 15a,b). As can be seen from the grain size distributions, a porous sample’s microstructure is represented by grains with the size varying between 0.8–6.1 μm with an average value of 2.4 μm. In the case of a dense sample, this range is 0.5–6.3 μm with an average value of 2.5 μm. The distributions in both cases are unimodal with one maximum localized in the range of 1.0–2.0 μm. We assume that the modulations used in dense sample production play the role of additional heat treatment (such as annealing or aging). In the case of porous samples’ manufacturing, the modulations were not used; instead, a remelting stage was introduced for each deposited and melted layer, which could cause additional grain growth after crystallization. It is known that Ti-based alloys produced by LPBF and other AM methods are characterized by grains with a size of tens of microns and nonequilibrium α′- and α″-martensite precipitations of 10–100 µm in length [57,58,78]. In our case, we observed mostly equiaxed grains with precipitations not exceeding 10 µm. Thus, we assume that the modulations and remelting stages could be used for sample production and provide conditions for additional grain growth of the main phase in comparison to LPBF mode without modulations or remelting stages. In addition, it should be noted that, in the case of the dense sample, there were α″-phase grains with a length not exceeding 700 nm and width of less than 100 nm (Figure 9), which are noticeably less than the 1000 nm length and 90–200 nm width (Figure 8) of α″-phase grains in porous LPBF sample. Formation of grains of the same phase with different sizes could also be due to the conditions of heat distribution realized in dense and porous samples by using different modes of modulations and remelting strategies.

**Figure 16:**
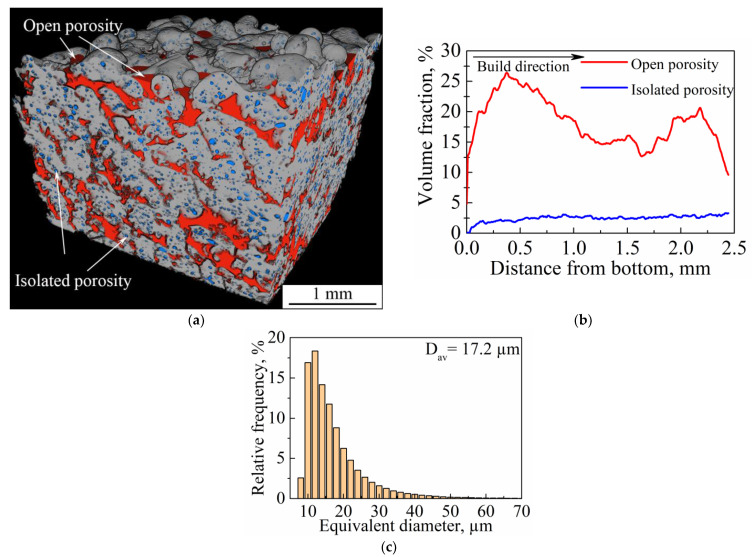
(**a**) Three-dimensional rendering of segmented voids in a section of a studied porous sample; (**b**) the volume fraction of open and isolated porosity as a function of distance from the sample’s bottom; (**c**) size distribution of isolated porosity (D_av_ is the average pore size).

The 3D internal structure of the porous sample was investigated by CT. As can be seen from the 3D rendering of segmented pores (Figure 16a), the main part of the inner structure is represented by an open interconnected system of pores, which is indicated in red; isolated porosity is colored in blue (Figure 16a,b). The volume fraction of isolated porosity is 2 vol. %., whereas total porosity is 20 vol. %. As can be seen from Figure 16b, isolated porosity (blue line) is uniformly distributed throughout the bulk of a sample. In addition, open porosity (Figure 16b, red line) is more developed near the sample’s bottom and top surfaces. This is because near the top surface isolated porosity is directly affected by the roughness of upper layers and would be transferred in the case of subsequent layer deposition. At the same time, the bottom surface is the one where the sample was cut from the substrate. Separation of the sample from the substrate led to the elimination of part of the pores’ boundaries. This distribution of open porosity near the substrate could also be connected to the high internal residual stresses, which are typical for the LPBF process. Its presence could cause the separation of the sample from the substrate. To estimate the size distribution of isolated porosity, the equivalent spherical diameter was used. As can be seen in Figure 16c, the isolated porosity size distribution has a unimodal character and the average pore size is 17.2 µm, which correlates with the results obtained from metallography images (Figure 15). However, the secant method allows only a few metallography images to be processed, whereas results obtained using CT are more reliable. As can be seen in Figure 16a, open pores can reach tens (crosswise) and hundreds (lengthwise) of microns in size. As reported in the work [17], pores with sizes of 20–500 µm are suitable for bone tissue regeneration and pores of 355–500 µm in size reveal good cell viability and adhesion.

**Figure 17:**
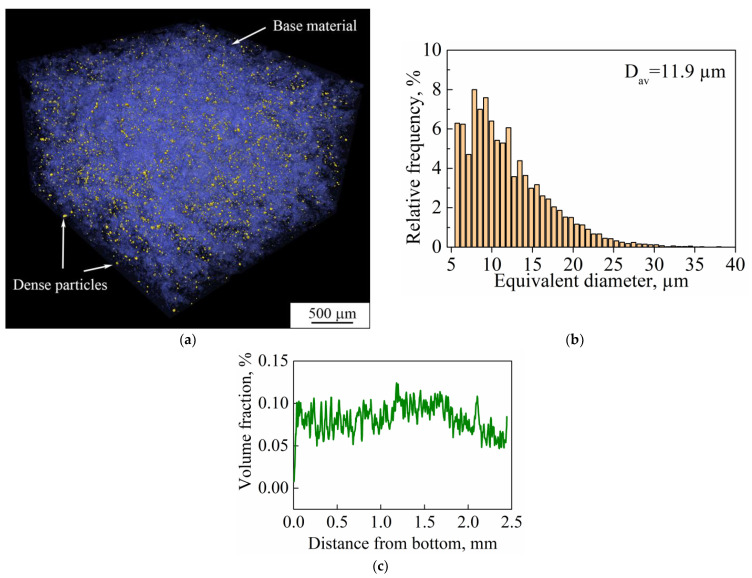
(**a**) Three-dimensional rendering of segmented phases (particles in yellow and base material in blue) in a porous sample; (**b**) the size distribution of dense particles; (**c**) distribution of the volume fraction of dense particles in the build direction of sample (D_av_ is the average particle size).

CT also allows the various phases in the bulk of the material to be separated according to the density values of those phases. Results of CT undertaken for the bulk porous samples showed that the studied material is represented by two phases. As can be seen in Figure 17a, the studied LPBF porous sample is represented by the base material and particles of unidentified second phase with higher density. Particles of the second phase have an irregular shape. Their size distribution is shown in Figure 17b. The distribution has a unimodal character and the average size of particles is 11 μm. Their distribution throughout the bulk of the sample is uniform (Figure 17c). The volume fraction of the second phase’s particles does not exceed 0.1%.

About 0.1 vol.% of unmelted Nb particles of 11 µm in size could provide a slight strengthening effect. However, the presence of pure Nb particles in the sample indicates the necessity of increasing the time of mechanical alloying. Another method to dispose of these particles was suggested in [45]. The authors observed inclusions of pure Nb particles throughout the bulk of LPBF samples made of Ti-6Al-7Nb and Ti-22Al-25Nb alloys. The authors note that annealing at 1350 °C for 2.5 h leads to Nb diffusion. Increasing the annealing time up to 3.5 h led to the total dissolution of those particles into the matrix. Thus, we can assume that subsequent annealing of the samples in our case could also provide a decrease in residual stress and the partial or total dissolution of Nb particles.

As described earlier, there is a significant difference between the density values of Ti and Nb, i.e., 4.51 g/cm^3^ and 8.57 g/cm^3^, respectively [83]. Thus, one can assume that unidentified particles could comprise traces of Nb. However, as stated above, for powder manufacturing we used niobium particles of an irregular shape with sizes not exceeding 25 μm (Figure 1). Because XRD profiles of pure β-Nb and β-TiNb-phases are almost identical, it is almost impossible to determine whether all pure niobium interacted with titanium and formed a solid solution; that is, unidentified dense particles could be represented by pure niobium particles that formed agglomerates in the process of mechanical alloying. As represented in Figure 6d,e, TEM maps of element distributions in nanostructured powder material used for LPBF and the production of studied samples show that there is a uniform distribution of Ti and Nb in the powder. However, TEM is a very local method and requires large statistics to be reliable. In addition, the dark-field image (Figure 6c) shows nanoscale grains of size not exceeding 90 nm. Thus, there could be agglomerates of pure Nb with a size of about 10–25 μm in the powder used for LPBF in a small amount. We do not assume that those particles could be pure Ti because it has a lower density than Nb.

**Figure 18:**
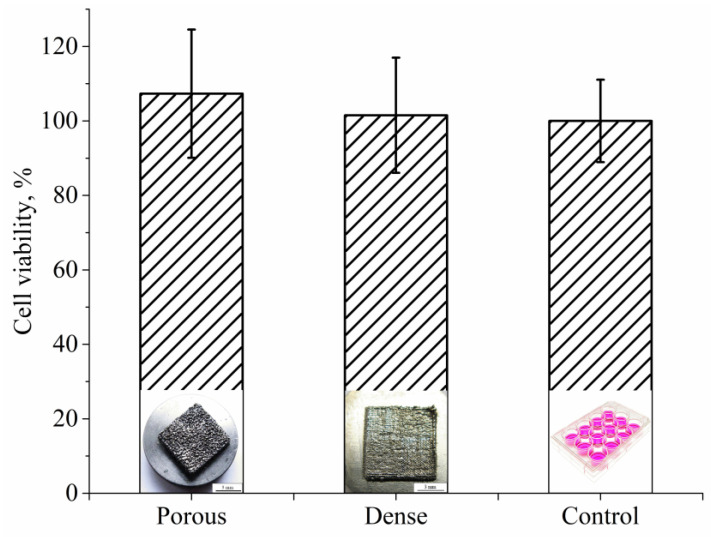
MG63 cell viability test for porous and dense Ti-Nb samples after 7 days of incubation.

The MTT test for cell viability assessment was performed after seven days of MG63 cell culturing in direct contact with both types of samples. The results of the viability study were compiled in Figure 18. Cell viability is expressed as a percentage of control mean viable cells on the control surface (cell culture plastic), showing increased cell viability of osteoblast-like cells growing on the porous LPBF sample when compared to the dense LPBF sample. Although the cell viability results are slightly higher for porous samples with respect to fully dense samples, the observed trend was not statistically significant (*p* < 0.05), and, therefore, no differences in cell viability could be observed between the two types of samples.

**Figure 19:**
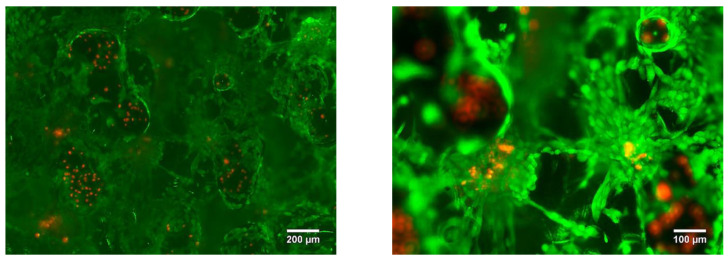
Porous Ti-45wt.% Nb sample LIVE/DEAD stained with Calcein AM/PI after 3 days of incubation.

The LPBF samples were tested for cytotoxicity using fluorescence microscopy. After three days of cell culture, no cytotoxic effect was found. It was revealed that the cells were migrating from the top layer of the sample into the bulk. In Figure 19 it is shown that the cells were spread into the pores, colonizing all of the available area provided by the sample surface and inner pore structure (Figure 19).

The cell attachment was high and resulted in adherence to most of the samples’ pointed tips and valleys without detaching. Homogeneous distribution of the cells in the bulk of the sample was not achieved solely by the cell migration, but also by the seeding method. Due to the open porosity of the LPBF sample (Figure 14), the cells that were transferred in the volume of medium slowly passed through the pores, filling the available volume. After three days of culture, the fluorescence images showed a similar cell density between fully dense and porous substrates.

**Figure 20:**
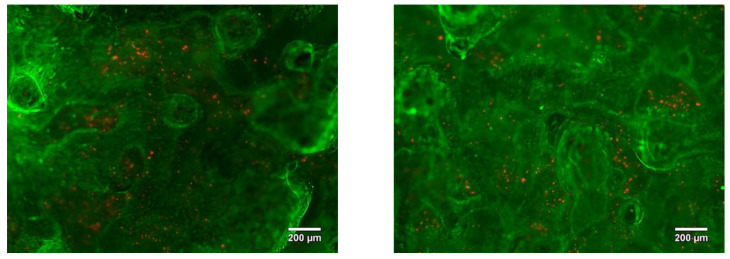
Porous Ti-Nb sample LIVE/DEAD stained with Calcein AM/PI after 7 days of incubation.

After seven days of incubation, the MG63 cells homogenously covered the whole visible surface of the porous LPBF sample, reaching 95% surface confluency. We suppose that the surface roughness and open porosity can support the cell adhesion and proliferation by imitating native extracellular matrix properties. Furthermore, with a longer culture times, cells were able to proliferate, showing an increased cell density over the entire surface. Indeed, on day seven the MG63 cells were covering the entire porous structure (Figure 20), even filling the pores attaching to pore walls and arriving at the edge of the samples, similarly to the findings presented in the paper by A. Civantos et al. [17].

**Figure 21:**
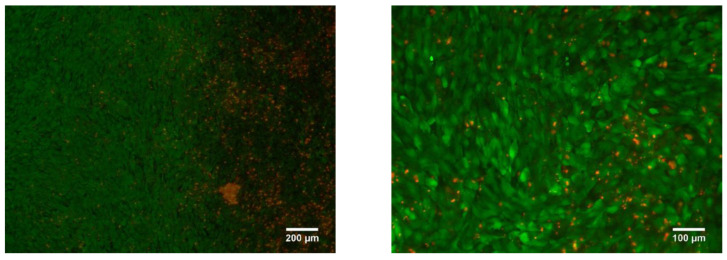
Dense Ti-Nb sample LIVE/DEAD stained with Calcein AM/PI after 7 days of incubation.

The other group of dense LPBF samples was also found to be non-cytotoxic. The rough surface of the sample allows cells to more easily adhere, resulting in a confluency coverage on the surface of close to 100% after seven days of incubation (Figure 21).

## 4. Conclusions

The production route for laser powder bed fusion manufacturing of porous and dense samples of low-modulus Ti-Nb alloy was shown in this study. The nanostructured powder material for LPBF was manufactured using an alternative approach to the commercially used spheroidization method. The study employed mechanical alloying, using commercially available pure powders of Ti and Nb in an AGO-2C planetary ball mill. The mechanical alloying allowed the production of impurity-free Ti-45 wt.% Nb nanostructured powders within only 15 min of mechanical alloying, with a particle size distribution in the range of 5–75 μm, which is desirable for the laser powder bed fusion process. Numerical simulation confirmed the nanoscale dimensionality of the produced powder material. Two different strategies for LPBF sintering were used, allowing us to manufacture bulk porous samples with a rough surface and dense samples characterized by a uniform, smooth surface. In both types of samples, uniaxial grains were visible, with identical microstructure features and phase composition for dense and porous samples. The inner structure of a porous sample was represented by an open interconnected system of pores. The volume fraction of isolated porosity was about 2 vol. %., whereas the total porosity was about 20 vol. %. Cell viability determination was performed in vitro after three and seven days of MG63 cell culturing in direct contact with the samples. With longer culture times, the MG63 cells were able to proliferate, showing an increased cell density over the entire surface. On day seven, the MG63 cells covered the entire porous structure, even filling the available open pores, and attaching to the pore’s walls. Both types of sample are not cytotoxic and could be used for further in vivo studies.

## Data Availability

All the data are reported in the paper directly.

## Supplementary Materials

The following are available online at https://www.mdpi.com/article/10.3390/nano11051159/s1, Gif-file S1: Strategies of remelting. The remelting–sintering strategy included the formation of each layer of the sample in two stages: sintering and remelting. At the sintering stage, a contour of a sintering zone was formed by a laser beam, following the predefined strategy. Inside the formed contour, powder was sintered along the straight lines, i.e., tracks, with a hatch distance of 0.4 mm. The same laser strategy was repeated with a 0.2 mm step from the already sintered tracks. The same sintering strategy was repeated in the vertical direction; subsequently, one layer was formed. To transform untreated, residual powder particles into a liquid state and ensure adequate sintering, and to reduce the level of internal stresses, the remelting stage was performed. The remelting stage was performed using a chess-like strategy for even and odd layers. The layer in one zone was divided into four regions, in which the remelting took place sequentially.
