# Supplementary figures and images for: Advances in Laser Additive Manufacturing of Ti-Nb Alloys: From Nanostructured Powders to Bulk Objects
Source: Nanomaterials (Basel). 2021 Apr 29;11(5):1159. doi: 10.3390/nano11051159 (PMC8145374; doi:10.3390/nano11051159)

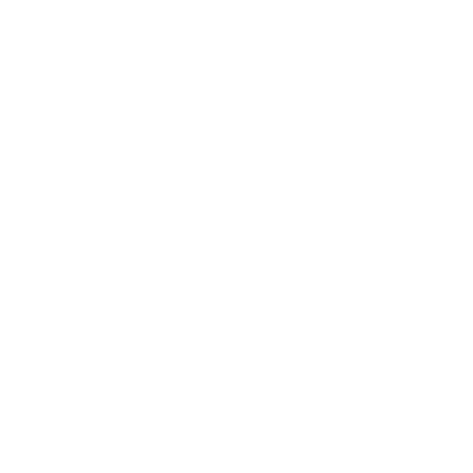

Supplement: Supplementary file 1 [file nanomaterials-11-01159-s001.zip › nanomaterials-1192855-supplementary.gif]
